# Optimal exercise parameters of Baduanjin for improving glycemic and lipid control in type 2 diabetes: a systematic review and meta-analysis

**DOI:** 10.3389/fendo.2026.1731466

**Published:** 2026-02-24

**Authors:** Shiying Zhang, Shun Fan, Hongyi Wang, Limeng Li, Kaihan Su, Lianjun Yin, Huanan Li, Jingui Wang, Xiaoyu Wang

**Affiliations:** 1 Department of Tuina, First Teaching Hospital of Tianjin University of Traditional Chinese Medicine, Tianjin, China; 2 National Clinical Research Center for Chinese Medicine, Tianjin, China; 3 Department of Rehabilitation, The Third Affiliated Hospital of Southern Medical University, Guangzhou, China; 4 Department of Rehabilitation, Guangdong Provincial Hospital of Traditional Chinese Medicine, Guangzhou, Guangdong, China; 5 The Postdoctoral Research Station, Guangdong Provincial Hospital of Chinese Medicine, Guangzhou, Guangdong, China

**Keywords:** Baduanjin, exercise parameters, glucose metabolism, lipid metabolism, meta-analysis, type 2 diabetes mellitus

## Abstract

**Background:**

This meta-analysis explores the optimal exercise parameters of Baduanjin for improving glucose and lipid metabolism in patients with type 2 diabetes mellitus (T2DM).

**Method:**

This meta-analysis was conducted according to the Preferred Reporting Items for Systematic Reviews and Meta-Analyses (PRISMA) guidelines. Eight databases were searched for randomized controlled trials (RCTs) published from their inception to April 30, 2025. Studies quality and risk of bias were assessed using the Cochrane Risk of Bias tool version 2 (RoB 2). Meta-analysis was performed using R software (version 4.4.2), primarily utilizing the meta and metafor packages. The certainty of evidence was assessed using the Grading Recommendations, Assessment, Development and Evaluation (GRADE) approach. The study protocol was registered in PROSPERO https://www.crd.york.ac.uk/PROSPERO/view/CRD42023480517.

**Results:**

This meta-analysis included 48 studies involving 3,699 participants. The findings demonstrate that Baduanjin significantly reduces fasting blood glucose (WMD = –0.78, 95% CI: –0.94 to –0.62, P < 0.0001, I**²** = 84.2%) and glycated hemoglobin (WMD = –0.67, 95% CI: –0.81 to –0.53, P < 0.0001, I² = 69.9%) in patients with T2DM, with a substantial effect on triglycerides (WMD = –0.37, 95% CI: –0.57 to –0.16, P < 0.0001, I² = 84.7%). Improvements in total cholesterol (WMD = –0.40, 95% CI: –0.56 to –0.25, P < 0.0001, I² = 86.6%) were comparatively modest. This study suggests potentially optimal exercise parameters for Baduanjin practice: 40–45 minutes per session, three times weekly, for a minimum duration of 24–48 weeks.

**Conclusion:**

Baduanjin significantly improves glucose and lipid metabolism in patients with T2DM. Our findings suggest that three sessions per week, each lasting 40–45 minutes, with an intervention duration of 24–48 weeks, may represent the optimal parameters for metabolic improvement.

**Systematic review registration:**

https://www.crd.york.ac.uk/prospero/, identifier Identifier CRD42023480517.

## 1 Introduction

Type 2 diabetes mellitus (T2DM) remains the predominant form of diabetes and continues to rise globally, imposing a substantial and growing health and economic burden. Recent global estimates and projections indicate that diabetes prevalence will keep increasing over coming decades, with most adult cases attributable to T2DM (1, 2). Cardiovascular disease is a major contributor to morbidity and mortality in people with T2DM, underscoring the need for comprehensive cardiometabolic risk reduction (3, 4). Accordingly, lifestyle modification— especially regular physical activity—remains a cornerstone of contemporary T2DM management; the American Diabetes Association recommends at least 150 minutes per week of moderate-to-vigorous aerobic activity (with resistance training as feasible for most adults) (5). Importantly, dysglycemia exists on a continuum, and prediabetes represents a critical and potentially reversible stage for preventing or delaying progression to T2DM. In recent years, traditional Chinese exercises (TCEs) have been increasingly evaluated in individuals with prediabetes. Recent systematic reviews and meta-analyses suggest that TCEs can improve glycemic indices and may confer modest benefits on lipid profiles in prediabetes populations. Moreover, a randomized controlled trial reported that one-year Baduanjin training improved multiple metabolic indices and reduced estimated cardiovascular risk in patients with prediabetes, with overall effects comparable to moderate-intensity aerobic training (6–8).

In this context, glycemic and lipid outcomes—commonly captured by fasting blood glucose (FBG), glycated hemoglobin (HbA1c), triglycerides (TG), and total cholesterol (TC)—are routinely used to evaluate metabolic benefits. Traditional Chinese exercise therapies (e.g., Baduanjin) have gained attention as accessible, low-to-moderate–intensity options, yet the optimal exercise parameters (frequency, session duration, and intervention period) for improving these metabolic outcomes remain unclear.

Regular physical activity is a cornerstone of type 2 diabetes mellitus (T2DM) management and is consistently recommended to improve glycemic control and reduce cardiometabolic risk (9–11). Evidence from controlled trials and meta-analyses indicates that structured exercise interventions can improve glycemic outcomes in patients with T2DM (10, 11).

In addition to conventional aerobic and resistance training, traditional TCEs such as Tai Ji Quan, Qigong, and Baduanjin have received increasing attention as feasible mind–body interventions for individuals with T2DM (12–15). Existing systematic reviews and meta-analyses suggest that these interventions can improve glycemic control (e.g., fasting blood glucose and HbA1c) and may also benefit lipid parameters in T2DM, although the magnitude of benefit varies across modalities and outcomes (13–15). TCEs comprise multiple modalities, which differ substantially in movement structure and training dose. Recent network meta-analyses that synthesize mixed mind–body/TCE interventions have highlighted between-modality variability and potential clinical heterogeneity, which may limit the translation of pooled findings into a clear, modality-specific dose–response exercise prescription. A recent Bayesian network and dose–response meta-analysis evaluated the optimal exercise dose for glycemic control in individuals with prediabetes across multiple exercise types, and classified traditional Chinese exercise–related interventions under a broad “mind–body training” (MBT) category (e.g., Tai Chi, Baduanjin, Yijinjing) (16). However, the authors noted heterogeneity across MBT modalities and the lack of sufficient high-quality, modality-specific trials to support practice-specific recommendations. In addition, that study primarily modeled dose using exercise volume (METs·min/week) and focused on HbA1c, without providing Baduanjin-specific temporal parameters (frequency, session duration, weekly practice time, and intervention cycle) or lipid outcomes in T2DM.To avoid between-modality mixing and to generate an actionable, modality-specific prescription, the present review isolates Baduanjin RCTs in patients with T2DM and synthesizes both glycemic and lipid outcomes while quantifying key temporal training parameters. Baduanjin is a relatively standardized eight-movement routine with low equipment requirements and is feasible for home- and community-based delivery, which may support long-term adherence in middle-aged and older adults (17). Compared with conventional higher-impact aerobic exercise (e.g., running), Baduanjin is a standardized low-to-moderate-intensity mind–body routine integrating slow coordinated movements with breathing regulation and focused attention/meditative components, and has been shown to be safe and feasible even in older adults with frailty or limited exercise tolerance (18). These mind–body breathing elements may help modulate autonomic balance, and long-term Baduanjin training has demonstrated cardiometabolic benefits comparable to moderate-intensity aerobic training in at-risk populations, supporting its suitability for T2DM management (19). Moreover, an expanding body of recent systematic reviews and trial protocols in people with type 2 diabetes mellitus (T2DM) provides sufficient evidence to explore frequency–duration–cycle parameters for metabolic outcomes, enabling a more actionable exercise prescription (20). Specifically, a Baduanjin-focused meta-analysis reported improvements not only in glycemic markers but also in lipid profiles (e.g., TC and TG) when Baduanjin was used as an adjunct to conventional therapy (21).

However, the current evidence base has not been adequately translated into an actionable exercise prescription. Prior studies and syntheses are frequently limited by small sample sizes, substantial heterogeneity, and inconsistent or incompletely reported intervention parameters (frequency, session duration, weekly practice volume, and intervention length) (12–15, 21). Importantly, most previous reviews did not explicitly quantify Baduanjin “dose” or examine potential dose–response relationships using parameter-oriented subgroup analyses or meta-regression, which hinders the identification of an evidence-informed optimal training protocol (12, 14, 15, 21). Therefore, the present systematic review and meta-analysis aims to update the evidence on both glycemic and lipid outcomes and to quantify key exercise parameters via subgroup analyses, thereby supporting a more precise and operationalizable Baduanjin exercise prescription for T2DM management (9, 12, 13).

Frequency, intensity, duration, and type of exercise are widely recognized as key parameters for assessing the effectiveness of exercise programs (22). Although the duration of Baduanjin practice is generally associated with improvements in glucose and lipid metabolism, the precise exercise prescription for diabetes management remains to be established. This study therefore aims to investigate the role of Baduanjin in improving specific blood glucose and lipid indices and to evaluate combinations of its parameters to identify the most effective Baduanjin protocol.

## 2 Methods

This study was conducted in strict accordance with the Preferred Reporting Items for Systematic Reviews and Meta-Analyses (PRISMA) guidelines and was registered with the International Prospective Register of Systematic Reviews (PROSPERO) under registration number CRD42023480517.

### 2.1 Literature search

Two reviewers (SYZ and SF) independently conducted a systematic literature search for randomized controlled trials (RCTs) investigating Baduanjin for T2DM. The search encompassed eight electronic databases: China National Knowledge Infrastructure (CNKI), Wanfang Database (WANFANG), VIP Database (VIP), China Biomedical Literature Service System (CBM), Embase, Web of Science, PubMed, and the Cochrane Library. We also examined clinical trial registries and grey literature sources. The search period extended from each database inception to April 30, 2025. Subject terms and keywords included “Baduanjin,” “eight section brocades,” “diabetes,” “prediabetes,” “diabetes patients,” “diabetes sufferers,” “type 2 diabetes,” “randomized controlled trials,” and “clinical trials.” We further manually screened the reference lists of all included studies. After duplicate removal, studies that clearly did not meet the inclusion criteria were excluded based on title and abstract screening. The full texts of the remaining articles were assessed for eligibility by two reviewers (SYZ and LML). Any disagreements were resolved through consultation with the third reviewer (XYW). The complete search strategy is available in **Supplementary Table 2**.

### 2.2 Inclusion and exclusion criteria

The inclusion criteria were (1): RCTs design (2); participants diagnosed with T2DM, irrespective of gender, age, or ethnicity (3); Baduanjin as an intervention in at least one group, with no restrictions on frequency, session duration, or program length (4); The control condition could be non-exercise/usual care (e.g., standard care, medication, health education, routine management, or daily activities) or an active exercise comparator (e.g., walking, aerobic exercise, resistance training, or other structured exercise programs). For analysis, control groups were *a priori* categorized as non-exercise versus active exercise comparators to enable sensitivity analyses assessing the impact of control group types on the pooled estimates (5); reporting of at least one outcome measure from FBG, HbA1c, TG, or TC.

Pharmacological therapy was permitted in the control condition because it represents routine standard care for T2DM and exercise interventions are typically evaluated as an adjunct to usual care. In this review, concomitant medication was considered a background co-intervention, and pooled effects were interpreted as the incremental benefit of adding Baduanjin beyond standard treatment. Medication-related information (e.g., drug use and co-interventions) was extracted when reported. However, medication regimens (drug classes, dosages, and treatment adjustments) were inconsistently reported across trials; therefore, subgroup analyses or meta-regression stratified by medication class/dose could not be performed.

The exclusion criteria were (1): studies not involving human subjects; (2) sample sizes smaller than 10 participants; (3) duplicate publications; and (4) studies from which relevant outcome data could not be obtained after (i) extracting data reported as Mean ± SD, (ii) transforming reported statistics (e.g., Median/IQR, Median/Range, five-number summary, or Mean with SE/CI) into Mean ± SD using established methods (23, 24), and (iii) attempting to contact the corresponding authors for missing numerical information.

### 2.3 Study selection and data extraction

Two reviewers (SYZ and SF) screened the literature using EndNote 21. When outcome data were missing, unclear, or not directly extractable, we contacted the corresponding authors of the original studies for clarification or to request additional information. If the required data could not be obtained, we attempted to extract data from figures/tables when feasible; otherwise, the outcome was excluded from quantitative synthesis. Two reviewers (SYZ and XYW) independently extracted data from the included studies, with any discrepancies resolved by the third reviewer. The extracted information encompassed the authors, publication year, participant characteristics (including age, gender distribution, and sample size), intervention group details (such as Baduanjin frequency, session duration, total weekly practice time, and intervention cycle), and outcome measures (including specific indicators and data). After extraction, reviewers cross-verified all entries in the data form for accuracy, and any disagreements were settled through discussion until a consensus was reached. For continuous outcomes, we preferentially extracted change-from-baseline data (mean change and its SD) for each group. If change scores were not reported, the mean change was obtained from baseline and post-intervention means. When the SD of change was unavailable, it was derived from baseline and post-intervention SDs using the within-person correlation coefficient (R) between baseline and post-intervention measurements. If R was not reported, we applied a prespecified plausible value (R = 0.50) and tested alternative plausible values (e.g., 0.25 and 0.75) in sensitivity analyses (25). When continuous outcomes were not reported as Mean ± SD, we attempted to convert the available summary statistics to Mean ± SD prior to exclusion. For studies reporting medians with interquartile ranges, ranges, or five-number summaries, Mean and SD were estimated using quantile-based estimation methods recommended for meta-analysis (23, 26). For studies reporting Mean with standard error (SE), SD was calculated as SD = SE × √*n*. When 95% confidence intervals (CI) were reported, SE was derived from the CI width using the appropriate t-quantile and then converted to SD. Studies were excluded only if conversion was infeasible due to insufficient reporting and unavailable author clarification (24, 27).

### 2.4 Risk of bias assessment

The methodological quality of the included studies was evaluated with the Cochrane Risk of Bias tool for randomized trials (RoB2). Two reviewers (HYW and SYZ) performed the assessments. The third reviewer (KHS) resolved any discrepancies that arose between the initial reviewers.

### 2.5 Data processing and statistical analysis

All statistical analyses were performed using R software (version 4.4.2), utilizing the meta (version 8.0-2) and metafor (version 4.6-0) packagesto assess pooled effect sizes via a random-effects model with the restricted maximum likelihood (REML) estimator. Continuous outcome measures (FBG, HbA1c, TG, and TC) were analyzed using Weighted Mean Difference (WMD) with 95% confidence intervals (CIs). WMD was selected as the effect size metric because all included studies reported objective biochemical indicators in identical units (or were converted to uniform units), thereby offering clinically interpretable results. The primary meta-analysis was conducted using change-from-baseline WMDs between groups for each outcome, with SDs of change handled as described above. To ensure comparability for WMD pooling, we harmonized units across studies before analysis. FBG, TG, and TC were converted to SI units (mmol/L) when necessary (glucose: mg/dL ÷ 18; TG: mg/dL ÷ 88.57; TC: mg/dL ÷ 38.67). HbA1c was analyzed in percent (%) as absolute percentage-point differences. The pooled effects were calculated automatically using the metacont function within the meta package. Heterogeneity among studies was assessed using the I^2^ statistic, where values >50% indicated substantial heterogeneity and ≤50% suggested low heterogeneity.

Subgroup analyses and comparisons—considering intervention frequency, session duration, weekly exercise volume, and intervention period—were performed to identify optimal exercise parameters for T2DM management. The subgroup cut-off values were prespecified *a priori* in the review protocol based on clinical relevance and commonly reported Baduanjin prescriptions. Subgroup categories were defined as follows (1): intervention frequency: 3 sessions/week, 5–7 sessions/week, and >7 sessions/week (2); session duration: 20 min, 30 min, 40–45 min, and 60–90 min (3); weekly exercise volume: 60–149 min/week, 150–210 min/week, 211–300 min/week, and >300 min/week (4); intervention period: 4–8 weeks, 12–14 weeks, and 24–48 weeks. These cut-offs were not modified after data extraction and no data-driven cut-point optimization was conducted. The cut-off values were determined based on two criteria (1): The natural clustering of intervention protocols identified during data extraction. Specifically, session durations in the included studies predominantly clustered at 40–45 minutes and 60–90 minutes, aligning with the standard Health Qigong Baduanjin protocol promulgated by the General Administration of Sport of China (28), where a complete session typically includes warm-up, one or two rounds of practice, and cool-down (2). International physical activity guidelines. The weekly exercise volume subgroup of 150–210 minutes was defined to align with the American Diabetes Association (ADA) and World Health Organization (WHO) recommendations, which advise a minimum of 150 minutes of moderate-intensity physical activity per week to optimize glycemic control (29, 30). Furthermore, intervention cycles were categorized into medium-term (12–14 weeks) and long-term (24–48 weeks) to assess the temporal stability of metabolic improvements.

We additionally conducted exploratory random-effects meta-regression (REML) to investigate potential sources of heterogeneity. Potential exercise-prescription moderators were prespecified according to the extracted parameters, including frequency, session duration, weekly volume, and intervention period. A moderator was entered into the meta-regression only when it was reported as a single numeric value and showed sufficient completeness across trials. Parameters reported as ranges (e.g., “3–7 sessions/week”) or as categorical descriptions were not converted into single values for regression to avoid introducing measurement error; these were instead examined using prespecified subgroup categories. In practice, meta-regression was feasible for intervention duration (weeks) only, due to incomplete or range-reported data for other parameters.

Because pharmacological therapy may influence glycemic and lipid outcomes, medication was considered a background co-intervention, and pooled estimates were interpreted as the incremental effect of Baduanjin beyond usual care. Owing to inconsistent reporting of medication regimens (e.g., drug class, dosage, and treatment adjustments), subgroup analyses or meta-regression stratified by medication class/dose could not be conducted.

Publication bias was evaluated through funnel plot inspection and Egger’s test, with a significance threshold of P < 0.05. If significant publication bias was detected, the Duval and Tweedie non-parametric “trim and fill” method was employed to estimate the number of missing studies and adjust the effect size to verify the robustness of the results. A leave-one-out sensitivity analysis was performed, where each study was sequentially excluded to assess the robustness of the overall effect. Given the clinical heterogeneity introduced by different comparator types and potential methodological quality concerns, we additionally conducted sensitivity analyses restricting the pooled estimates to two scenarios: (1) trials using non-exercise controls only (i.e., excluding active exercise comparators); and (2) trials excluding those with a high risk of bias. This was performed for each primary outcome (FBG, HbA1c, TG, and TC).

### 2.6 Certainty of evidence

The quality of evidence was assessed using the Grading of Recommendations, Assessment, Development, and Evaluation (GRADE) approach. RCTs are initially considered high-quality evidence (31). The quality rating is then adjusted according to specific criteria: factors such as limitations in study design, inconsistency of results, indirect evidence, imprecision, or publication bias each warrant a one-level downgrade, potentially to very low quality. Conversely, upgrading may occur for reasons such as a large magnitude of effect or a dose-response gradient. Any disagreements during assessment were resolved through discussion among all reviewers.

## 3 Result

### 3.1 Literature screening

**Figure 1:**
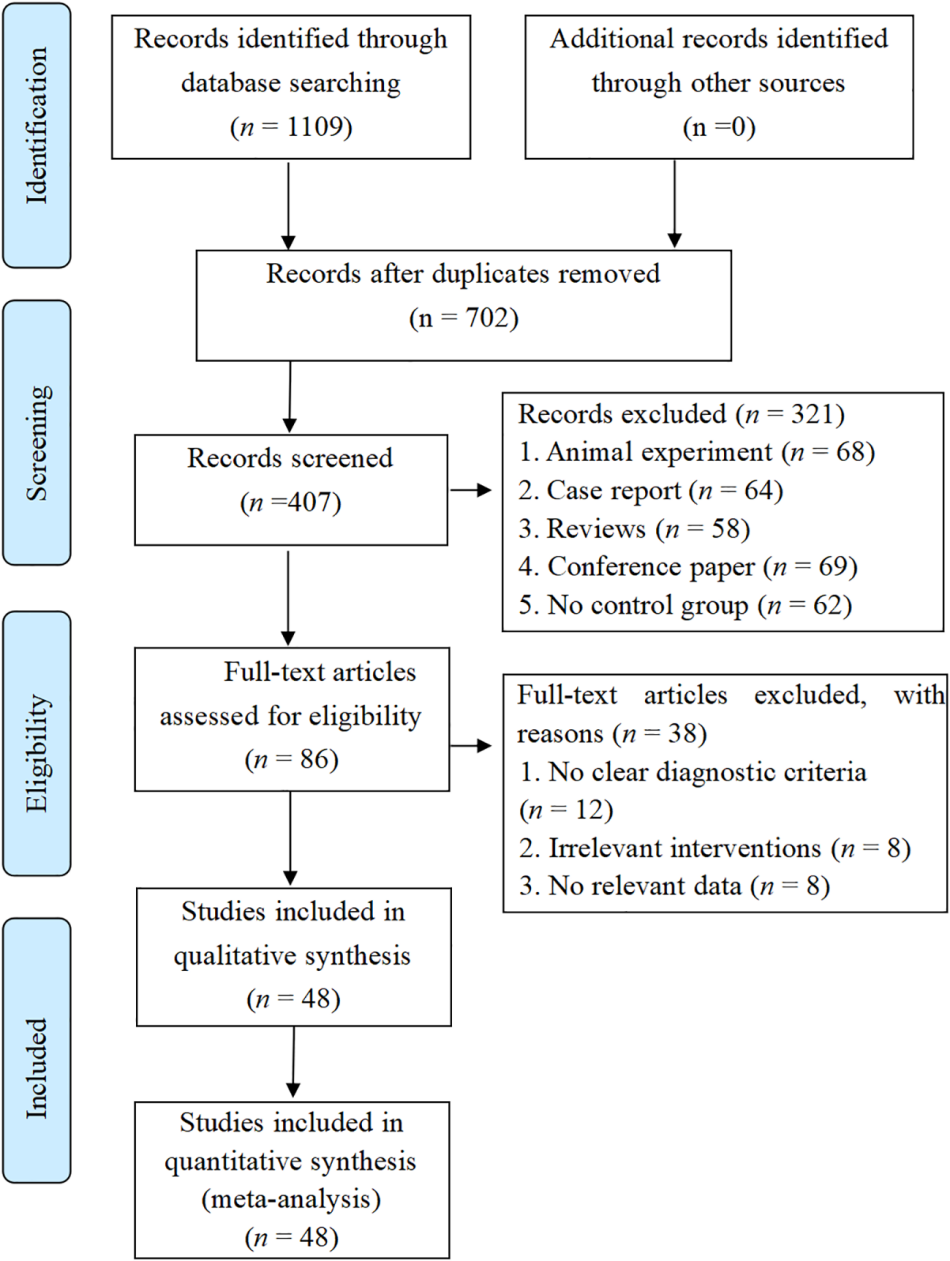
Study flowdiagram.

Our comprehensive search of the eight specified databases initially yielded 1109 articles. Following duplicate removal, 407 records remained. After screening titles and abstracts, we excluded 321 articles that failed to meet the inclusion criteria, resulting in 86 articles for full-text assessment. A detailed review of these full texts led to the exclusion of 38 additional articles. Ultimately, 48 RCTs were included in the final analysis (**Figure 1**).

### 3.2 Literature characteristics

After search screening, 48 studies involving 3699 participants met the inclusion criteria and were included in this meta-analysis (32–79). Among these, 42 studies evaluated the effect of Baduanjin on FBG in patients with T2DM (32–49, 51–55, 57–60, 62, 63, 67–75, 77–79). Another 41 studies assessed its effect on HbA1c levels (32–40, 42–47, 50–59, 61, 62, 64–67, 69–77, 79), while 24 studies examined its impact on TG levels (32–35, 38, 39, 42–47, 51–55, 59, 67, 69, 72, 73, 75, 79), and 22 studies investigated its effect on TC levels (32–35, 38, 39, 43, 44, 47–49, 51–55, 67, 69, 72, 73, 76, 79). The age of the patients predominantly ranged from 50 to 70 years, while one study did not report the age of its participants. In most studies, the number of male and female participants was approximately balanced at a ratio of 1:1, eight studies did not explicitly state the male-to-female ratio, and nine included two or more control groups (**Table 1**).

**Table 1:** Characteristics of included RCTs.

Study	Sample size (T/C)	Age (T/C)	Gender (M/F)	Experimental group method	Control group method	Intervention (Baduanjin) programs				Outcomes
						Cycle (w)	Frequency (n/w)	Duration per time (min)	Duration per week (min/w)	
Jiang Y (2019) (32)	84 (42,42)	T: 68.61 ± 8.22 C:67.42 ± 9.16	T: 25/17 C: 23/19	Baduanjin + C	High-quality nursing care	12	14	20	280	①②③④
Qi YH (2023) (33)	30(15,15)	T: 52.4 ± 7.35 C: 53.5 ± 5.63	T: 9/6 C: 8/7	Baduanjin + C	Pharmacotherapy	12	5	60	300	①②③④
Fang CP (2014) (34)	89(30,30,29)	T: 56.62 ± 8.85 C1:57.13 ± 9.26 C2:58.24 ± 8.91	T: 16/14 C1: 17/13 C2:15/14	Baduanjin + C	C1:Health education C2: Health education + walk	12	10	30	300	①②③④
Liu T (2018) (35)	40(20,20)	T: 57.2 ± 5.4 C: 57.2 ± 5.4	/	Baduanjin	Daily activity	24	6	90	540	①②③④
Zhou T (2014) (36)	25(13,12)	/	T: 2/11 C: 4/8	Baduanjin	Health education	12	7	30	210	①②
Zhang X (2011) (37)	30(15,15)	T: 39-69 C: 39-69	T: 6/9 C: 7/8	Baduanjin + C	Regular examinations	24	7	60	420	①②
Wang YG (2007) (38)	79(40,39)	T:57.8 ± 7.5 C: 56.5 ± 6.9	T: 28/12 C: 25/14	Baduanjin + C	Pharmacotherapy	24	7	60	420	①②③④
Li XH (2009) (39)	79(40,39)	T: 57.8 ± 7.5 C: 56.5 ± 6.9	T: 28/12 C: 25/14	Baduanjin + C	Pharmacotherapy	24	7	60	420	①②③④
Lin YF (2013) (40)	38(19,19)	T: 64.5 ± 11.5 C: 60.8 ± 12.2	T: 12/7 C: 10/9	Baduanjin + C	Pharmacotherapy	24	7	45	315	①②
Zu SL (2021) (41)	68(34,34)	T: 55.5 ± 10.9 C: 55.8 ± 12.3	T: 19/15 C: 18/16	Baduanjin + C	Pharmacotherapy	4	7	/	/	①
Yang YQ (2023) (42)	70(35,35)	T: 68.96 ± 3.77 C:69.44 ± 3.12	T: 24/11 C: 21/14	Baduanjin + C	High-quality nursing care	/	14	20	280	①②③
Huang RC (2011) (43)	60(30,30)	T: 57.8 ± 7.5 C: 56.5 ± 6.9	T: 18/12 C: 20/10	Baduanjin + C	Pharmacotherapy	24	7	60	420	①②③④
Li Y (2017) (44)	86(43,43)	T: 44.32 ± 5.07 C: 42.91 ± 6.1	T: 23/20 C: 22/21	Baduanjin	Pharmacotherapy	12	4-7	30	120-210	①②③④
Zhou LB (2011) (45)	126(63,63)	T: 67.4 ± 9.23 C:68.13 ± 10.64	T: 32/31 C: 31/32	Baduanjin	Daily activity	12	7	30	210	①②③
Guan YX (2012) (46)	79(39,40)	T: 59.2 ± 8.8 C: 58.7 ± 8.3	T: 18/21 C: 20/20	Baduanjin + C	Pharmacotherapy + Daily activity	12	7	60	420	①②③
Yi WM (2019) (47)	26(13,13)	T: 53.54 ± 8.21 C: 54.11 ± 9.06	T: 6/7 C: 5/8	Baduanjin	Pharmacotherapy	12	5-7	90	450-630	①②③④
Duan JH (2012) (48)	200(100,100)	T: 47 ± 7 C: 45 ± 9	T: 80/20 C: 78/22	Baduanjin	Daily activity	8	7	20	140	①④
Pan HS (2008) (49)	48(24,24)	T: 47 ± 7 C: 45 ± 9	T: 14/10 C: 16/8	Baduanjin + C	Pharmacotherapy	24	14	45	630	①④
Liu HH (2014) (50)	40(20,20)	T: 57 ± 7 C: 55 ± 9	T: 9/11 C: 8/12	Baduanjin + C	Pharmacotherapy	24	5	30	300	②
Zhao TQ (2022) (51)	24(12,12)	T: 67.43 ± 1.77 C:67.78 ± 1.93	/	Baduanjin + C	Pharmacotherapy	12	7	60	420	①②③④
Zhu YF (2023) (52)	20(10,10)	T: 53.8 ± 2.44 C: 55.7 ± 2.21	T: 4/6 C: 7/3	Baduanjin + C	Pharmacotherapy	14	6	60	360	①②③④
Huang L (2021) (53)	80(40,40)	T: 53.25 ± 2.2 C:53.22 ± 2.21	T: 25/15 C: 20/20	Baduanjin	Walk	12	5-7	60	300-420	①②③④
Song ZQ (2022) (54)	20(10,10)	T: 65.9 ± 4.25 C: 66.4 ± 4.69	T: 4/6 C: 4/6	Baduanjin + C	Pharmacotherapy	12	7	60	420	①②③④
Li FX (2020) (55)	80(40,40)	T: 57.63 ± 6.59 C:57.23 ± 6.23	T: 22/18 C: 21/19	Baduanjin + C	Resistance exercise	12	6	20	120	①②③④
Gong L (2021) (56)	60(30,30)	T: 62.64 ± 8.42 C:62.35 ± 8.12	T: 19/11 C: 18/12	Baduanjin + C	Pharmacotherapy	12	7	/	/	②
Zhang SJ (2015) (57)	108(45,45,18)	T: 57 ± 2.4 C1: 55.4 ± 1.7 C2:58.2 ± 1	T: 25/20 C1: 23/22 C2:10/8	Baduanjin	C1:Daily activity C2: Pharmacotherapy	14	7	30	210	①②
Peng RD (2019) (58)	65(33,32)	T: 60.88 ± 4.59 C:62.31 ± 4.96	/	Baduanjin + C	Pharmacotherapy	24	10	/	/	①②
Wei P (2023) (59)	100(50,50)	T:49.62 ± 12.78 C:52.03 ± 10.59	T: 22/23 C: 21/24	Baduanjin + C	Pharmacotherapy	24	7	30	210	①②③
Peng DZ (2015) (60)	139(69,70)	/	/	Baduanjin + C	Pharmacotherapy	24	7	30	210	①
Yang QJ (2017) (61)	105(53,52)	T: 52.4 ± 2.3 C: 50.3 ± 3.4	T: 26/27 C: 25/27	Baduanjin + C	Pharmacotherapy	12	7	60	420	②
Zheng LH (2022) (62)	95(47,48)	T: 58.7 ± 11.3 C: 59.1 ± 10.9	T: 25/23 C: 28/19	Baduanjin + C	Nursing care	12	7	30	210	①②
Shi LM (2019) (63)	57(28,29)	T: 68.21 ± 4.21 C:68.59 ± 4.65	T: 12/16 C: 13/16	Baduanjin	Walk	12	5	30	150	①
Liu Y (2012) (64)	69(33,36)	T: 62.64 ± 5.98 C:65.64 ± 8.38	T: 14/19 C: 14/22	Baduanjin + C	Nursing care	12	6-10	20	120-200	②
Huo R (2010) (65)	69(33,36)	T: 62.64 ± 5.98 C:65.64 ± 8.38	T: 14/19 C: 14/22	Baduanjin + C	Nursing care	12	5-7	30	150-210	②
Yang L (2023) (66)	79(40,39)	T: 67.5 ± 3.1 C: 67.4 ± 2.8	T: 13/27 C: 18/21	Baduanjin + C	Daily activity	12	3	60	180	②
Li XL (2023) (67)	238(119,119)	T: 69.61 ± 0.92 C:69.84 ± 1.37	T: 64/55 C: 61/58	Baduanjin + C	Pharmacotherapy + Resistance exercise	12	3	/	/	①②③④
Li WH (2019) (68)	141(49,45,47)	T: 56.9 ± 3.94 C1:56.2 ± 4.67 C2:56.34 ± 4.03	T: 15/34 C1: 18/27 C2:14/33	Baduanjin	C1:Resistance exercise C2: Pharmacotherapy	36	3-7	20	60-140	①
Ma YF (2021) (69)	67(35,32)	T: 60.4 ± 8.6 C: 60.9 ± 5.2	T: 15/20 C: 14/18	Baduanjin	Pharmacotherapy + Daily activity	12	5	30	150	①②③④
Wang XL (2019) (70)	100(50,50)	T:61.67 ± 10.51 C:61.76 ± 10.54	T: 25/25 C: 24/26	Baduanjin + C	Pharmacotherapy	12	5	30	150	①②
Luo F (2021) (71)	80(40,40)	T:66.54 ± 10.32 C:67.32 ± 9.46	T: 24/16 C: 27/13	Baduanjin + C	Pharmacotherapy	12	3	40	120	①②
Yang H (2018) (72)	50(17,15,18)	T: 57.28 ± 4.86 C1:62.36 ± 5.39 C2:56.91 ± 6.32	/	Baduanjin	C1:Walk C2: Health education	24	5	30	150	①②③④
Li P (2017) (73)	44(12,11,11,10)	T: 62.17 ± 5.06 C1:64.27 ± 4.45 C2:58.91 ± 8.78 C3:62.4 ± 6.06	/	Baduanjin	C1:Resistance exercise C2: Walk C3: Health education	24	10	/	/	①②③④
Chen XY (2014) (74)	29(10,9,10)	T: 60.00 ± 5.23 C1:63.33 ± 7.78 C2:63.70 ± 5.40	/	Baduanjin	C1:Health education C2: Health education + Walk	24	5	/	/	①②
He K (2019) (75)	75(24,27,24)	T:51.83 ± 14.32 C1:49.36 ± 11.54 C2:51.59 ± 11.46	T: 12/12 C1: 14/13 C2:13/11	Baduanjin	C1:Walk + Resistance exercise C2: Daily activity	48	7	30-60	210-420	①②③
Li ZB (2013) 76)	142(50/48/44)	T: 50.42 ± 9.68 C1:51.62 ± 7.83 C2:54.21 ± 9.47	T: 23/27 C1: 22/26 C2:20/24	Baduanjin	C1:Daily activity C2: Pharmacotherapy	12	7	30	210	①②④
Zhou JQ (2012) (77)	18(5,6,7)	T: 59 ± 7.6 C1: 61 ± 5.5 C2:66.4 ± 4.4	/	Baduanjin	C1:Walk C2: Pharmacotherapy	12	15-21	/	/	①②
Chu LL, 2024 (78)	100(50,50)	T: 55.92 ± 6.03 C:56.39 ± 5.87	T: 32/18 C: 27/23	Baduanjin	Nursing care	14	7	30	210	①
Yang MC (2012) (79)	59(29,30)	T: 60.83 ± 5.2 C: 58.1 ± 7.92	T: 9/20 C: 15/15	Baduanjin + C	Pharmacotherapy	24	7-14	30	210-420	①②③④

T, Treatment; C, control; w, week; min, minute; ①, FBG; ②, HbA1C; ③, TG; ④, TC

### 3.3 Risk bias

**Figure 2:**
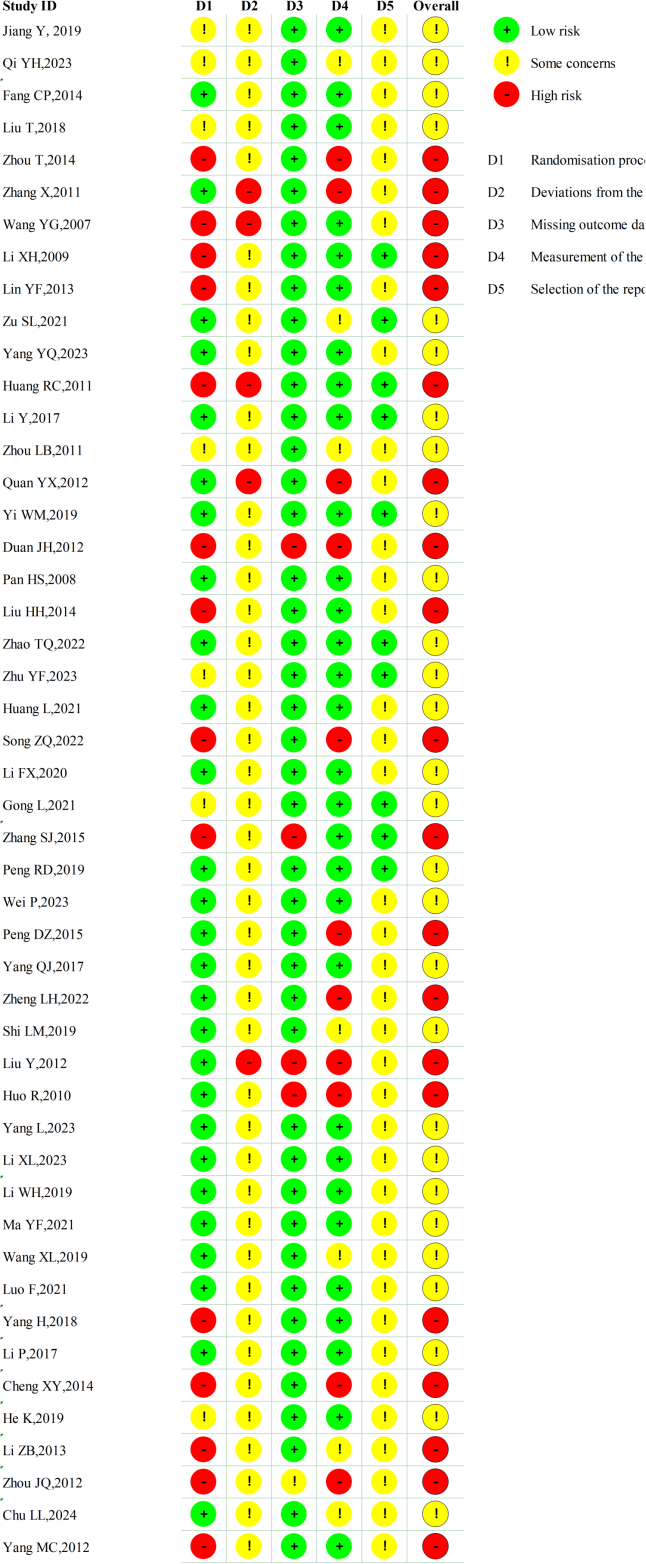
Risk of bias of RCTs: risk of bias graph.

**Figure 3:**
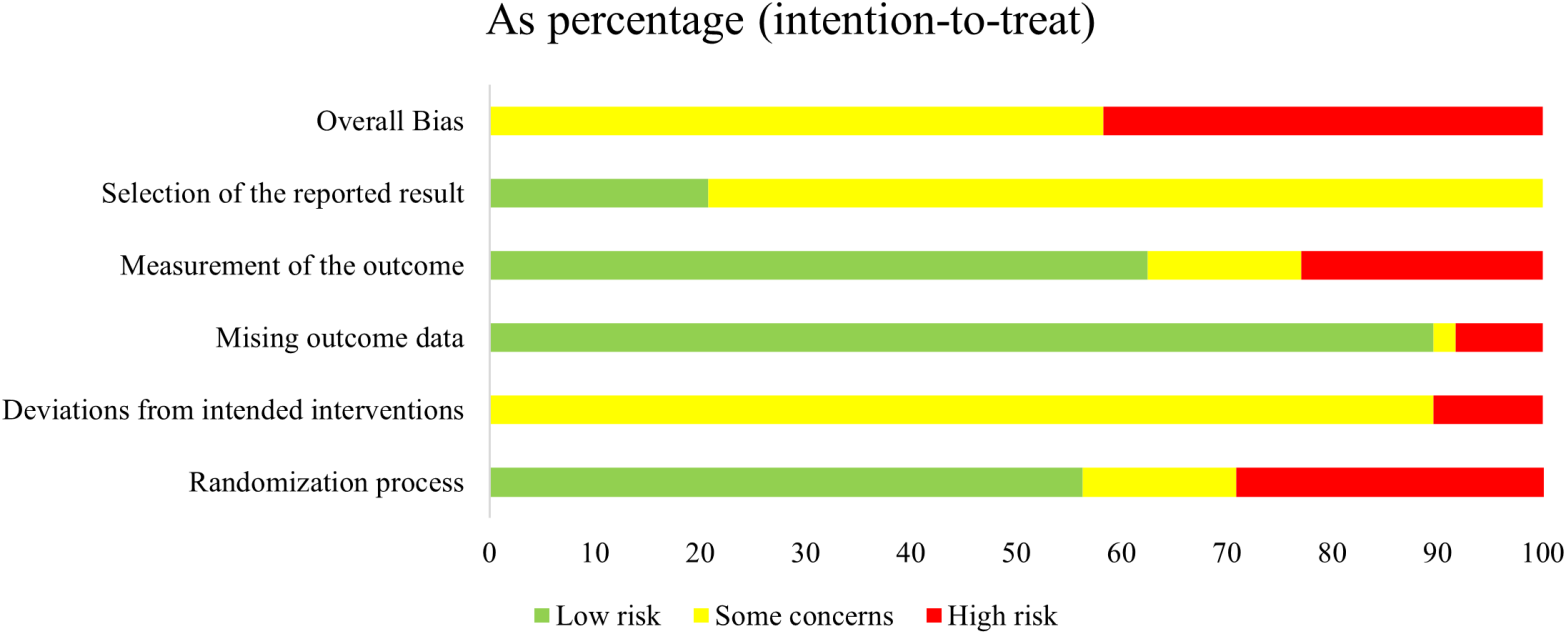
Risk of bias of RCTs: risk of bias summary.

According to the RoB2 assessment, 28 studies (32–35, 41, 42, 44, 45, 47, 49, 51–53, 55, 56, 58, 59, 61, 63, 66–71, 73, 75, 78) were rated as having some concerns and 20 studies (36–40, 43, 46, 48, 50, 54, 57, 60, 62, 64, 65, 72, 74, 76, 77, 79) as high risk. Due to the nature of the Baduanjin intervention, participant and personnel blinding could not be implemented in any of the trials, thereby representing a major source of bias and contributing to the overall low quality of evidence (**Figures 2**, **3**).

### 3.4 Meta-analysis

#### 3.4.1 FBG

**Figure 4:**
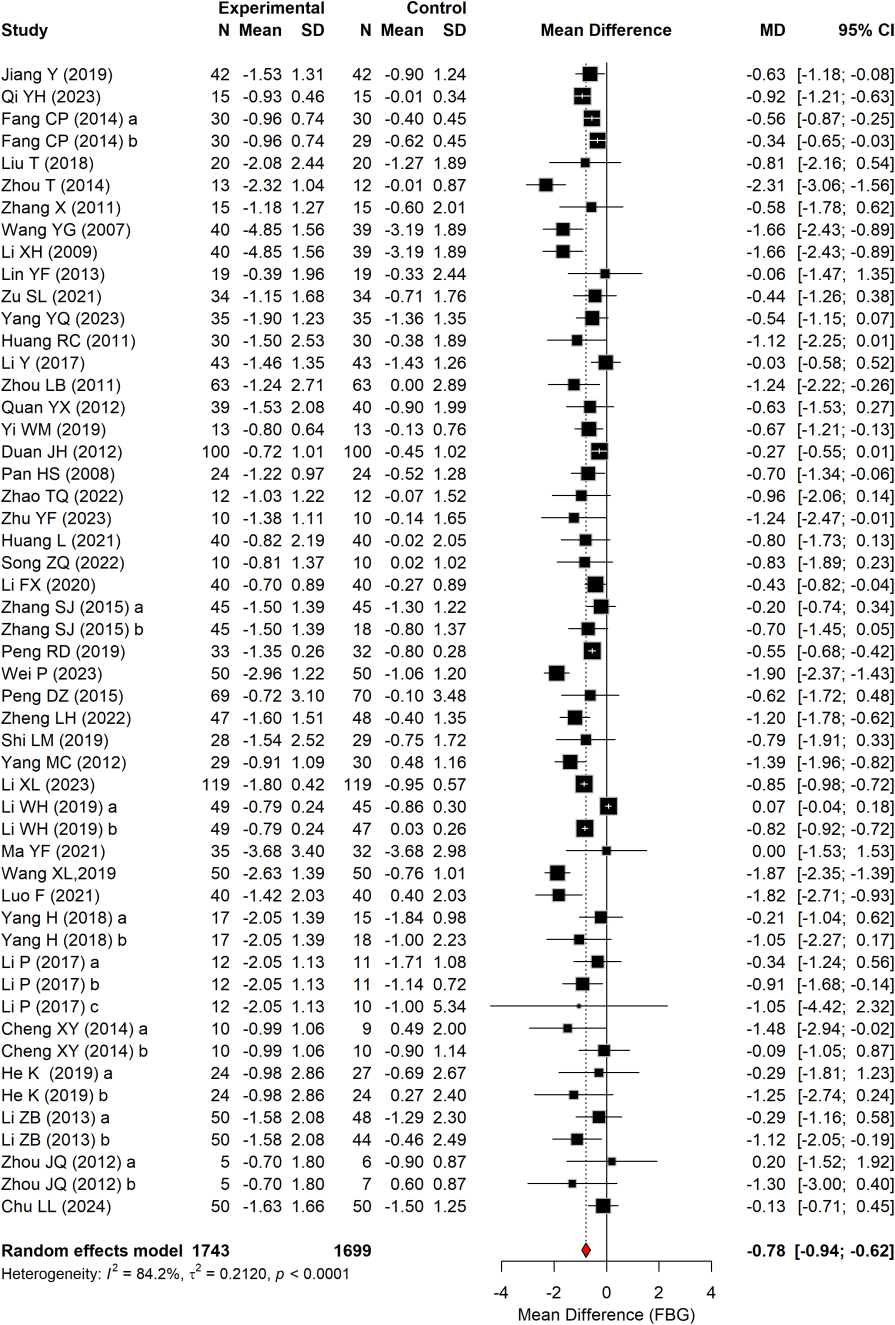
Forest plot of FBG according to the comparison of Baduanjin vs. control.

**Figure 4** presents the effect of Baduanjin on FBG in patients with T2DM, based on 42 RCTs comprising 3,442 participants. A significant reduction in FBG was observed in the Baduanjin group compared with the control group (WMD = –0.78 mmol/L, 95% CI: –0.95 to –0.61, P < 0.0001, I^2^ = 84.2%).

#### 3.4.2 HbA1c

**Figure 5:**
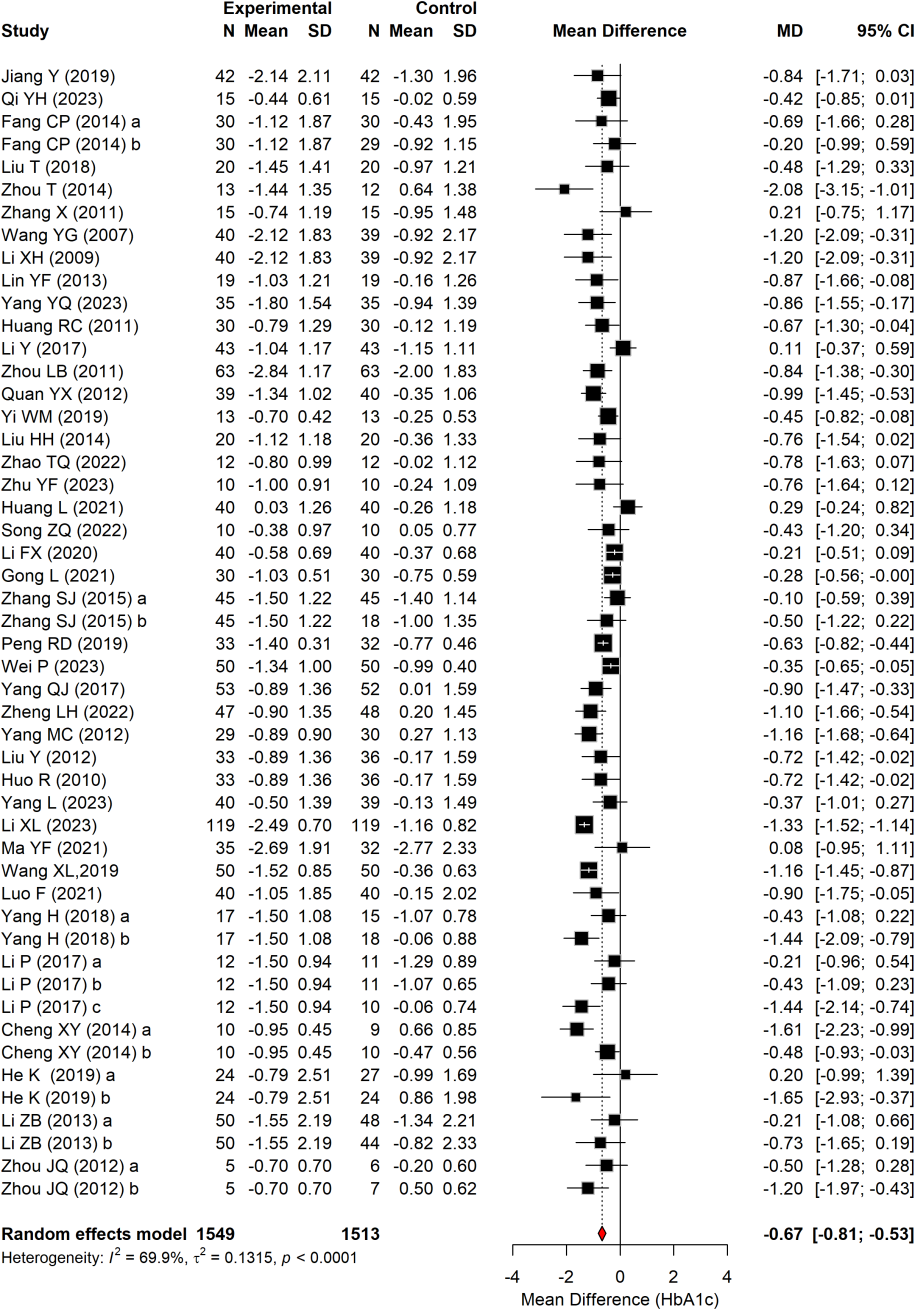
Forest plot of HbA1C according to the comparison of Baduanjin vs. control.

**Figure 5** presents the effect of Baduanjin on HbA1c in patients with T2DM, summarizing data from 41 RCTs comprising 3,062 participants. A significant reduction in HbA1c levels was observed in the Baduanjin group compared to the control group (WMD = –0.67 percentage points, 95% CI: –0.81 to –0.54, P < 0.0001, I^2^ = 69.9%).

#### 3.4.3 TG

**Figure 6:**
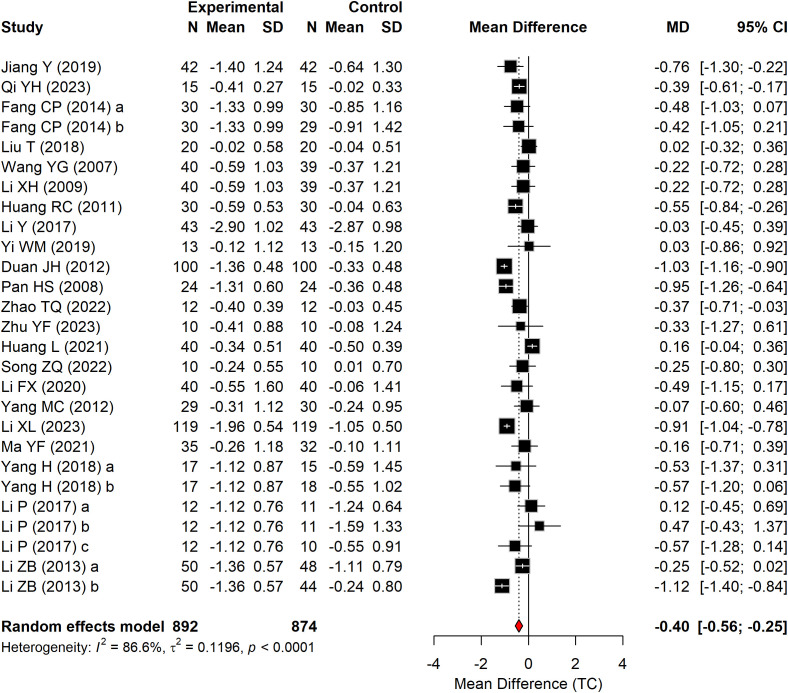
Forest plot of TG according to the comparison of Baduanjin vs. control.

**Figure 6** presents the effect of Baduanjin on TG levels in patients with T2DM, based on 24 RCTs comprising 1,800 participants. A significant reduction in TG was observed in the Baduanjin group compared with the control group (WMD = –0.37 mmol/L, 95% CI: –0.55 to –0.18, P < 0.0001, I^2^ = 84.7%).

#### 3.4.4 TC

**Figure 7:**
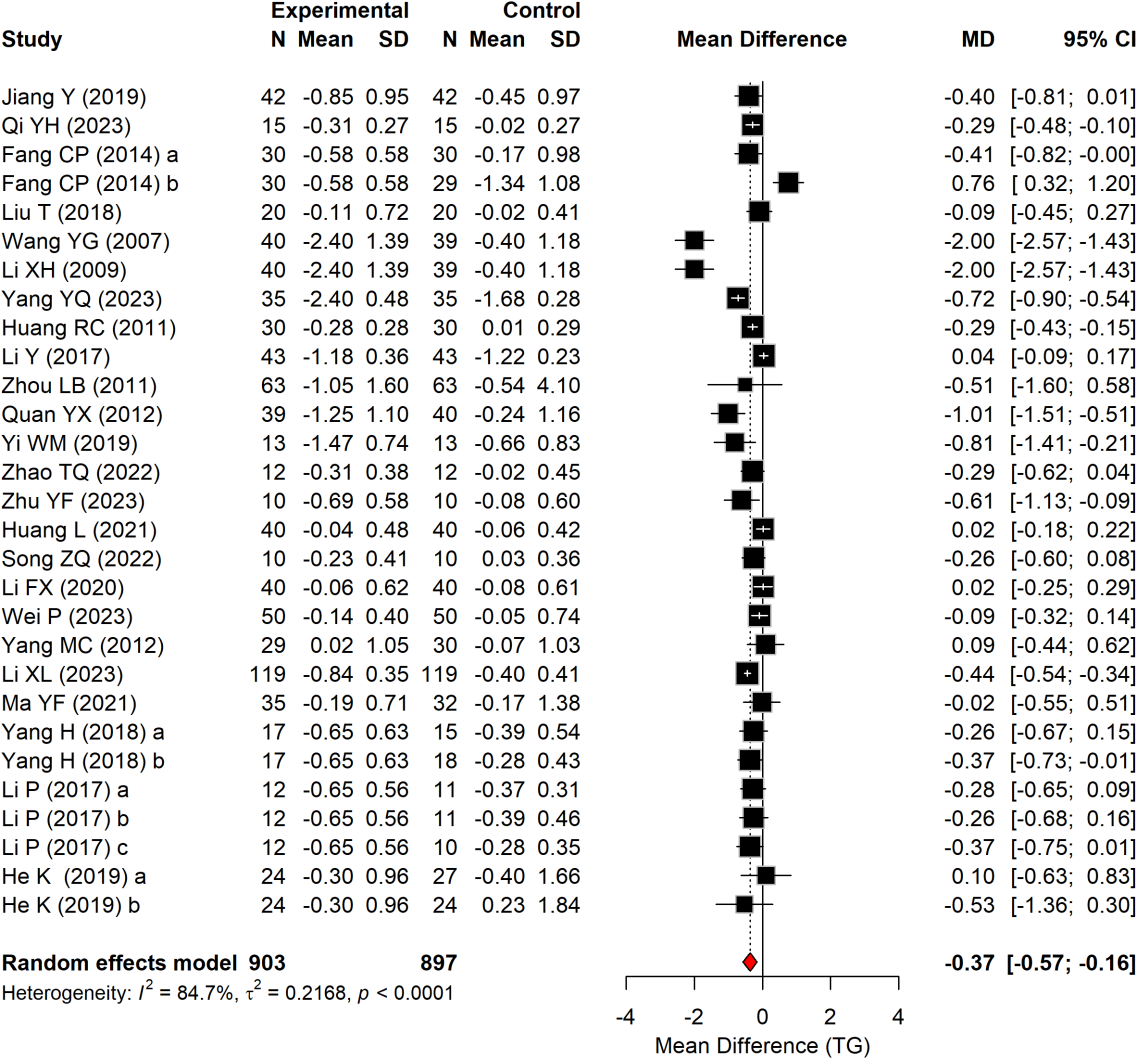
Forest plot of TC according to the comparison of Baduanjin vs. control.

**Figure 7** presents the effect of Baduanjin on TC in patients with T2DM, based on 22 RCTs comprising 1,766 participants. A significant reduction in TC was observed in the Baduanjin group compared with the control group (WMD = –0.40 mmol/L, 95% CI: –0.56 to –0.24, P < 0.0001, I^2^ = 86.6%).

### 3.5 Subgroup analysis

The **Table 2** summarizes the subgroup analyses of Baduanjin exercise parameters on glycemic and lipid outcomes in T2DM. For transparency, the number of included studies (S) and participants (N) contributing to each subgroup are reported in **Table 2**.

**Table 2:** Effect of different Baduanjin exercise parameters on the indicators of glucose and lipid metabolism in T2DM patients.

FBG						HbA1C					TG					TC				
Frequency (n/w)		S	N	I^2^	P		S	N	I^2^	P		S	N	I^2^	P		S	N	I^2^	P
3	-1.23(-2.16 to -0.30)	2	318	77.70%	0.0094	-0.93(-1.54 to -0.32)	3	397	76.8%	0.0028	-0.44(-0.54 to -0.34)	1	238	–	<0.001	-0.91(-1.04 to -0.78)	1	238	–	<0.001
5~7	-0.84(-1.06 to -0.62)	36	2398	69.90%	<0.001	-0.62(-0.79 to -0.45)	11	2108	65.5%	<0.001	-0.43(-0.68 to -0.18)	20	1162	84.3%	<0.001	-0.37(-0.56 to -0.18)	19	1150	88.4%	<0.001
>7	-0.57(-0.67 to -0.46)	12	536	14.10%	<0.001	-0.74(-0.96 to -0.52)	35	488	27.7%	<0.001	-0.22(-0.53 to 0.09)	8	1800	83.6%	0.162	-0.40(-0.72 to -0.08)	8	378	66.1%	0.0155
Duration per time (min)																				
20	-0.42(-0.72 to -0.12)	6	624	96.40%	0.0065	-0.41(-0.94 to -0.15)	4	303	40.6%	0.0067	-0.37(-0.82 to 0.07)	3	234	89.9%	0.0995	-1.00(-1.12 to -0.87)	3	364	38%	<0.001
30	-0.86(-1.19 to -0.53)	19	1495	82.10%	<0.001	-0.68(-0.81 to -0.55)	18	1308	69.3%	<0.001	-0.06(-0.29 to 0.17)	9	624	64.4%	0.6093	-0.41(-0.68 to -0.14)	9	590	73.6%	0.0028
40~45	-0.94(-1.88 to -0.00)	3	166	65.5%	0.0489	-0.88(-1.46 to -0.31)	2	118	0%	0.0027	–	0	0	–	–	-0.95(-1.26 to -0.64)	1	48	–	<0.001
60-90	-0.97(-1.16 to -0.77)	12	567	0%	<0.001	-0.54(-0.70 to -0.38)	14	751	42.8%	<0.001	-0.66(-1.06 to 0.25)	11	537	89.1%	0.0015	-0.21(-0.40 to -0.03)	10	458	62.3%	0.0223
Duration per week(min/w)																				
60~149	-0.56(-1.09 to -0.03)	5	550	97.3%	0.0372	-0.44(-1.07 to 0.20)	2	160	55.7%	0.1781	0.02(-0.25 to 0.29)	1	80	–	0.8844	-0.86(-1.35 to -0.37)	2	280	59.4%	<0.001
150~210	-0.96(-1.37 to -0.55)	15	1052	78%	<0.001	-0.74(-1.00 to -0.48)	14	904	67.5%	<0.001	-0.18(-0.35 to -0.02)	5	360	0%	0.0324	-0.37(-0.75 to -0.00)	3	134	0%	0.0474
211~300	-0.62(-0.78 to -0.46)	5	303	46.3%	<0.001	-0.57(-0.85 to -0.29)	6	343	0%	<0.001	-0.23(-0.71 to 0.25)	5	303	89.7%	0.3455	-0.44(-0.62 to -0.26)	4	233	0%	<0.001
>300	-0.93(-1.18 to -0.69)	13	624	0%	<0.001	-0.59(-0.77 to -0.42)	13	680	46.5%	<0.001	-0.70(-1.15 to -0.26)	10	507	90.1%	0.0021	-0.28(-0.53 to -0.04)	10	476	79.3%	0.0219
Training cycle(w)																				
4~8	-0.55(-1.57 to 0.47)	3	314	39%	0.1476	-0.57(-1.13 to -0.01)	1	105	–	0.0443	–	0	0	–	–	–	0	0	–	–
12~14	-0.61(-0.80 to -0.43)	34	2089	84.2%	<0.001	-0.49(-0.63 to -0.35)	35	2123	67.7%	<0.001	-0.15(-0.31 to 0.00)	18	897	58.9%	0.0502	-0.40(-0.56 to -0.24)	17	803	52.9%	<0.001
24~48	-0.84(-1.09 to -0.58)	26	1590	92.8%	<0.001	-0.78(-1.00 to -0.56)	21	1068	56.9%	<0.001	-0.50(-0.93 to -0.07)	12	590	85.4%	0.0275	-0.26(-0.44 to -0.08)	11	490	13.2%	0.0100

FBG, Fasting blood glucose; HbAlc, glycosylated hemoglobin; TG, Triglyceride; TC, total cholesterol.

#### 3.5.1 Intervention frequency

For FBG, the optimal intervention effect occurred at 3 sessions per week (WMD = −1.23 mmol/L, 95% CI: −2.16 to −0.30, I^2^ = 77.7%), whereas a moderate effect was observed with 5−7 sessions per week (WMD = −0.84 mmol/L, 95% CI: −1.06 to –0.62, I^2^ = 69.9%); the differences among the three groups were statistically significant (P < 0.05). For HbA1c, the greatest reduction was also achieved with 3 sessions weekly (WMD = −0.93 percentage points, 95% CI: −1.54 to −0.32, I^2^ = 76.8%), and group differences were significant (P < 0.05).

In lipid metabolism, no significant between-subgroup differences were observed for TG. The 3 sessions/week subgroup showed the largest numerical reduction (WMD = −0.44 mmol/L, 95% CI: −0.54 to −0.34), but this estimate was based on only one study (*n* = 238) and should be interpreted cautiously. For TC, subgroup differences were statistically significant (P < 0.05). The 3 sessions/week subgroup showed a larger reduction in TC (WMD = −0.91 mmol/L, 95% CI: −1.04 to −0.78); however, this estimate was likewise derived from only one study (n = 238) and warrants cautious interpretation. In comparison, the ≥7 sessions/week subgroup also showed a reduction, but of smaller magnitude (WMD = −0.40 mmol/L, 95% CI: −0.72 to −0.08; I^2^ = 66.1%).

#### 3.5.2 Duration of each session

Subgroup comparisons revealed significant differences (P < 0.05) for FBG, HbA1c, and TC, but not for TG. The most substantial reduction in FBG occurred with intervention durations of 60–90 minutes per session (WMD = −0.97 mmol/L, 95% CI: −1.16 to −0.77, I^2^ = 0%). For HbA1c, the greatest effect was observed with sessions lasting 40–45 minutes (WMD = −0.88 percentage points, 95% CI: −1.46 to −0.31, I^2^ = 0%). For TG, the 60–90 minutes subgroup showed the largest numerical reduction (WMD = −0.66 mmol/L, 95% CI: −1.06 to 0.25; I^2^ = 89.1%), although the confidence interval crossed the null. In contrast, 20–minute sessions were most effective for TC (WMD = −1.00 mmol/L, 95% CI: −1.12 to −0.87, I^2^ = 38%).

#### 3.5.3 Weekly exercise volume

Subgroup analyses indicated that the total weekly duration of Baduanjin practice significantly influenced FBG and TC outcomes among patients with T2DM (P < 0.05), although no significant differences were observed for HbA1c or TG subgroups. A weekly intervention duration of 150–210 minutes produced the greatest reduction in blood glucose levels (FBG: WMD = −0.96 mmol/L, 95% CI: −1.37 to −0.55, I^2^ = 78%; HbA1c: WMD = −0.74 percentage points, 95% CI: −1.00 to −0.48, I^2^ = 67.5%). For TG, the largest reduction was observed in studies prescribing >300 min/week (WMD = −0.70 mmol/L, 95% CI: −1.15 to −0.26). In contrast, the optimal improvement in TC was achieved with a weekly duration of 60–149 minutes (WMD = –0.86 mmol/L, 95% CI: –1.35 to –0.37, I^2^ = 59.4%).

#### 3.5.4 Intervention duration

For FBG and TG, larger reductions were generally observed in longer-duration subgroups, with the 24–48 week subgroup showing the greatest reductions (FBG: WMD = –0.84 mmol/L, 95% CI: –1.09 to –0.58, I^2^ = 92.8%; TG: WMD = –0.50 mmol/L, 95% CI: –0.93 to –0.07, I^2^ = 85.4%). HbA1c also exhibited its greatest reduction during the 24–48 weeks period (WMD = –0.78 percentage points, 95% CI: –1.00 to –0.56, I^2^ = 56.9%). In contrast, the largest effect on TC occurred earlier, at 12–24 weeks (WMD = –0.40 mmol/L, 95% CI: –0.56 to –0.24, I^2^ = 52.9%). Statistically significant differences between subgroups were observed for FBG, HbA1c, and TC (P < 0.05), whereas no such differences were found for TG.

### 3.6 Meta-regression analysis

A univariable meta-regression was performed to examine whether intervention duration (weeks) moderated the effect sizes of Baduanjin across outcomes. The **Supplementary Table 3** and **Supplementary Figure 1** indicated that intervention duration was a significant source of heterogeneity for HbA1c (P = 0.016, R^2^ = 16.60%). The regression coefficient was −0.0162 (95% CI: −0.0294 to −0.0031), suggesting that for each additional week of intervention, the weighted mean difference (WMD) in HbA1c between the Baduanjin and control groups decreased by a further 0.0162 percentage points, indicating significantly greater improvement with longer intervention duration. For triglycerides (TG), the meta-regression showed a trend toward statistical significance (P = 0.067), implying that a longer Baduanjin intervention may be associated with greater TG improvement. However, no significant linear association between intervention duration and effect size was observed for fasting blood glucose (FBG; P = 0.173) or total cholesterol (TC; P = 0.283), suggesting that improvements in these outcomes may not be solely dependent on extending the intervention period.

### 3.7 Level of evidence

Among the four outcome measures in this study, three (FBG, HbA1c, and TG) were supported by low–quality evidence. One measure (TC) was supported by very low–quality evidence, which limits the reliability of its effect estimate and warrants cautious interpretation (**Table 3**).

**Table 3:** Evidence quality assessment according to GRADE.

Baduanjin for T2DM						
Patient or population: patients with T2DM Settings: Intervention: Baduanjin						
Outcomes	Illustrative comparative risks* (95% CI)		Relative effect (95% CI)	No of Participants (studies)	Quality of the evidence (GRADE)	Comments
	Assumed risk	Corresponding risk				
	Control	Baduanjin				
FBG		The mean FBG in the intervention groups was 0.78 standard deviations lower (0.95 to 0.61 lower)		3442 (42 studies)	⊕⊕⊝⊝ low^1,2^	WMD -0.78 (-0.95 to -0.61)
HbA1C		The mean HbA1C in the intervention groups was 0.67 standard deviations lower (0.81 to 0.54 lower)		3062 (41 studies)	⊕⊕⊝⊝ low^1,2^	WMD -0.67 (-0.81 to -0.54)
TG		The mean TG in the intervention groups was 0.37 standard deviations lower (0.55 to 0.18 lower)		1800 (24 studies)	⊕⊕⊝⊝ low^1,2^	WMD -0.37 (-0.55 to -0.18)
TC		The mean TC in the intervention groups was 0.40 standard deviations lower (0.56 to 0.24 lower)		1766 (22 studies)	⊕⊝⊝⊝ very low^1,2,3^	WMD -0.40 (-0.56 to -0.24)

*The basis for the assumed risk (e.g. the median control group risk across studies) is provided in footnotes. The corresponding risk (and its 95% confidence interval) is based on the assumed risk in the comparison group and the relative effect of the intervention (and its 95% CI).

CI, Confidence interval.

GRADE Working Group grades of evidence

High quality: Further research is very unlikely to change our confidence in the estimate of effect.

Moderate quality: Further research is likely to have an important impact on our confidence in the estimate of effect and may change the estimate.

Low quality: Further research is very likely to have an important impact on our confidence in the estimate of effect and is likely to change the estimate.

Very low quality: We are very uncertain about the estimate.

^1^imprecision of results due to sparse data (total population size was less than 500)

^2^substantial heterogeneity

^3^publication bias

### 3.8 Publication bias

**Figure 8:**
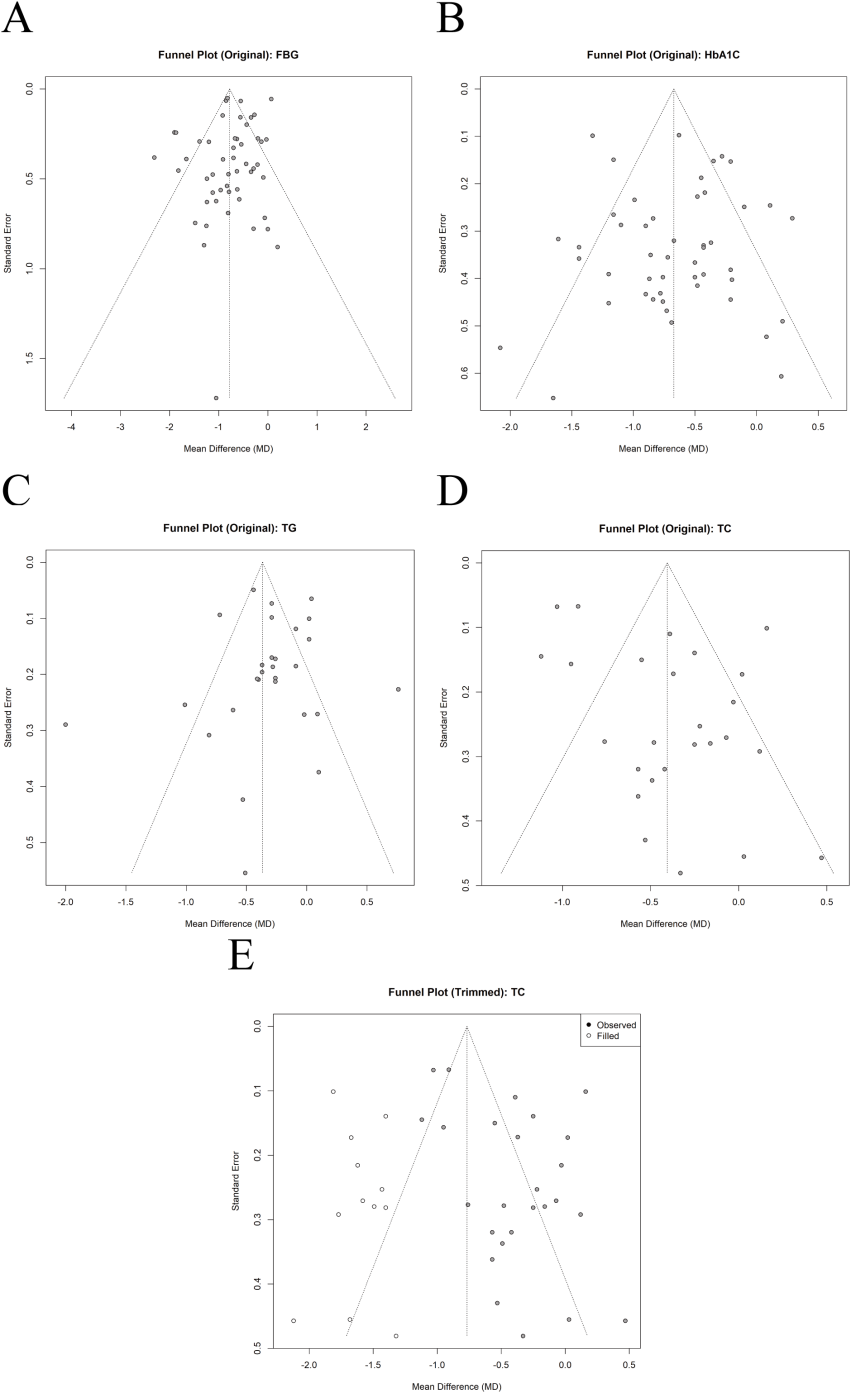
Publication bias: **(A)** Funnel Plot for FBG , **(B)** Funnel Plot for HBA1C, **(C)** Funnel Plot for TG, **(D)** Funnel Plot for TC, **(E)** Funnel Plot (Trimmed) for TC.

Funnel plots were generated to assess the risk of publication bias for outcomes reported in more than 10 studies (see **Figure 8**, **Supplementary Table 4**). The funnel plots for FBG, HbA1c, and TG were largely symmetrical. Egger’s test indicated no significant publication bias for FBG (P = 0.09), HbA1c (P = 0.60), or TG (P = 0.45), a finding consistent with the visual inspection of the funnel plots. In contrast, significant publication bias was detected for TC (P = 0.01) using both Egger’s test and funnel plot inspection. Given this bias, a trim-and-fill analysis was performed. The analysis imputed 13 potentially missing studies, resulting in an adjusted WMD of –0.77 (95% CI: –0.98 to –0.56). The adjusted result remained statistically significant (P < 0.0001), suggesting that the improvement in TC associated with Baduanjin is robust despite the potential publication bias.

### 3.9 Sensitivity Analysis

Sensitivity analyses were conducted to assess the robustness of the findings using three distinct approaches.

First, the “leave-one-out” method confirmed consistent outcomes across all four measures, indicating that no single study disproportionately influenced the pooled estimates (**Supplementary Figure 2**).

Second, to address the potential impact of study quality, we performed an analysis excluding studies rated as “high risk of bias.” The results indicated that Baduanjin remained effective in improving metabolic indicators even when analyzing only studies with lower risk of bias. Specifically, significant reductions were maintained for FBG (WMD = –0.73, 95% CI: –0.94 to –0.52), HbA1c (WMD = –0.58, 95% CI: –0.74 to –0.42), TC (WMD = –0.32, 95% CI: –0.54 to –0.10), and TG (WMD = –0.37, 95% CI: –0.60 to –0.13) (**Supplementary Figure 3**).

Third, to reduce clinical heterogeneity attributable to active exercise comparators, we performed sensitivity analyses restricting comparisons to non-exercise control groups only. The pooled effects remained statistically significant and were directionally consistent with the primary analyses: FBG (MD = −0.88, 95% CI: −1.06 to −0.69, P < 0.001), HbA1c (MD = −0.72, 95% CI: −0.86 to −0.57, P < 0.001), TC (MD = −0.45, 95% CI: −0.62 to −0.28, P < 0.001), and TG (MD = −0.47, 95% CI: −0.71 to −0.23, P < 0.001) (**Supplementary Figure 4**). These findings collectively suggest that the observed benefits of Baduanjin are robust, regardless of study quality or comparator type.

## 4 Discussion

### 4.1 Main findings

This meta-analysis synthesizing 48 RCTs (n = 3,699) indicates that Baduanjin yields statistically significant improvements in glycemic and lipid outcomes in T2DM, including FBG, HbA1c, TG, and TC, compared with control conditions. Importantly, subgroup analyses suggest that a dose pattern of 40–45 min per session, ~3 sessions per week, sustained for 24–48 weeks may be associated with the most consistent metabolic improvements.

In exploratory univariable meta-regression, Baduanjin intervention duration (weeks) emerged as a significant moderator for HbA1c, indicating that longer Baduanjin programs were associated with larger HbA1c reductions. This is clinically plausible because HbA1c reflects average glycemic exposure over approximately 8–12 weeks and may therefore be more responsive to the cumulative effects of sustained Baduanjin practice. By contrast, Baduanjin intervention duration showed only a borderline trend for TG and no significant linear association for FBG or TC, suggesting that changes in these outcomes may be influenced by additional factors (e.g., differences in comparators, baseline metabolic profiles, adherence, or medication adjustments) rather than the duration of Baduanjin alone.

Recent syntheses focusing on Baduanjin (or mind–body exercise sets that include Baduanjin) generally support beneficial effects on glycemic control and cardiometabolic markers, but they also highlight substantial limitations, including heterogeneous training prescriptions, variable comparators, and trial-level risks of bias (20). In addition, earlier reviews rarely quantified “dose” in a manner that supports clinical prescription (80). Our findings extend this literature by providing parameterized estimates of training dosage most consistently associated with improvements across glucose and lipid outcomes, thereby refining Baduanjin-specific exercise prescription rather than reiterating generic exercise recommendations.

Mechanistically, Baduanjin is characterized by coordinated low-to-moderate intensity movements integrated with breath regulation, relaxation, and meditative attention, which may engage metabolic benefits through mind–body mechanisms beyond energy expenditure alone. First, mind–body practice may facilitate autonomic nervous system (ANS) rebalancing, often indexed by heart rate variability (HRV). In T2DM, impaired autonomic regulation (e.g., reduced HRV) is clinically relevant, and exercise training has been shown to improve HRV parameters in T2DM populations, supporting ANS modulation as a plausible pathway (81).

Second, the meditative components of Baduanjin (paced breathing, mindful attention, relaxation) may reduce chronic stress and hypothalamic-pituitary-adrenal (HPA) axis activation; cortisol biology is closely linked to insulin resistance and cardiometabolic risk. Recent evidence indicates physical activity interventions can lower cortisol and improve sleep-related outcomes (82, 83), and mind–body exercise reviews propose neuroendocrine and inflammatory modulation (including stress-related pathways) as key contributors to health effects (84).

Third, emerging Baduanjin-related trials in other clinical contexts suggest improvements in HRV and recovery-related autonomic indices, which is consistent with a parasympathetic-enhancing, stress-reducing profile (19).

Nevertheless, we emphasize that many Baduanjin RCTs in T2DM do not routinely measure mechanistic biomarkers (e.g., HRV, cortisol), so the mechanistic discussion should be interpreted as biologically plausible and literature-supported, but not definitive for the included trials.

### 4.2 Strengths

This study has several strengths. First, to our knowledge, it is among the most comprehensive syntheses aiming to parameterize Baduanjin dosage (frequency, session duration, weekly volume, and intervention cycle) for glycolipid outcomes in T2DM, rather than merely estimating overall efficacy. Second, we used RoB 2 for risk-of-bias assessment and GRADE to rate the certainty of evidence, alongside sensitivity analyses, providing a transparent appraisal of evidence strength. Third, by integrating subgroup analyses across multiple dosage dimensions, we offer clinically interpretable, testable hypotheses for Baduanjin prescription in future high-quality trials.

### 4.3 Clinical implications

Our findings support Baduanjin as a feasible adjunct to usual care for adults with T2DM, particularly for individuals who prefer culturally grounded, low-barrier mind–body activity. Given its integration of coordinated movement, breath regulation, and meditative attention, Baduanjin may be especially relevant when clinicians aim to improve metabolic control alongside stress-related barriers to self-management (e.g., poor sleep, anxiety, low exercise adherence).

Importantly, the dosage pattern identified in this review should be interpreted as a preliminary suggestion rather than a definitive clinical prescription. These subgroup definitions were selected to mirror clinically actionable components of a Baduanjin prescription (sessions/week, min/session, min/week, and intervention duration in weeks) and to align with commonly used protocol structures and physical activity guidance, thereby improving the translational interpretability of the subgroup findings. Subgroup analyses across multiple dosage dimensions suggested that approximately 40–45 minutes per session, around three sessions per week, sustained for 24–48 weeks may be associated with the most consistent improvements across glycemic and lipid outcomes. Consistent with this, exploratory univariable meta-regression indicated that longer Baduanjin intervention duration (weeks) was associated with greater HbA1c reduction, supporting the notion that sustained practice may be particularly relevant for longer-term glycemic control. In practice, clinicians should individualize recommendations according to age, comorbidity burden, baseline activity, and safety considerations, with ongoing monitoring of metabolic response and appropriate treatment adjustments as needed.

### 4.4 Limitations

Several limitations warrant caution. First, overall trial quality was suboptimal: no included RCT was rated as “low risk of bias” under RoB 2, and blinding is inherently difficult for exercise interventions, increasing risks of performance and detection bias. Second, heterogeneity was substantial across outcomes, reflecting differences in participant characteristics, co-interventions, adherence, and outcome measurements. Third, evidence certainty was limited (low for FBG/HbA1c/TG and very low for TC), and publication bias was detected for TC, reducing confidence in the pooled estimates.

Fourth, ‘optimal parameters’ were inferred from prespecified subgroup analyses and exploratory univariable meta-regression. Given the potential for false positives arising from multiple comparisons, these findings should be interpreted with caution as hypothesis-generating rather than definitive prescriptions, especially where subgroup cells were supported by few studies. Notably, meta-regression was limited to Baduanjin intervention duration (weeks) because training frequency, session duration, and weekly practice time were frequently reported as ranges (e.g., “3–7 sessions/week”) or categorical/qualitative descriptions and were incompletely reported across trials; therefore, these parameters were not converted into single continuous values for regression and were primarily evaluated using prespecified subgroup categories. Consequently, residual heterogeneity is likely influenced by other unmeasured or incompletely reported factors, and our ability to examine multiple Baduanjin dose moderators simultaneously was limited.

Fifth, our reliance on prespecified subgroup categories to summarize Baduanjin “dose” may not fully capture the complexity of continuous exposure–outcome relationships. Methodological guidance highlights that continuous variables often have non-linear associations, and categorization can lead to loss of information and potentially misleading inferences about dose–response patterns (85, 86). Therefore, our subgroup-derived “optimal parameters” should be interpreted as hypothesis-generating rather than precision prescriptions. Future trials and meta-analyses with more granular reporting (or individual participant data) are needed to evaluate potential non-linear dose–response relationships using flexible approaches (e.g., splines or fractional polynomials).

Sixth, reporting of key training characteristics (notably intensity, supervision, and adherence) was inconsistent, limiting dose–response inference. Finally, background pharmacotherapy (drug class/dose changes) may confound metabolic outcomes; although many trials aimed for stable medication regimens, reporting was incomplete and residual confounding cannot be ruled out. In addition, comparator types varied across trials (non-exercise/usual care vs. active exercise), which may contribute to clinical heterogeneity; therefore, we provided comparator-restricted sensitivity analyses to improve interpretability. Future large, rigorously designed trials should standardize Baduanjin protocols, report intensity/adherence comprehensively, and incorporate mechanistic endpoints (e.g., HRV, cortisol, inflammatory markers) to clarify pathways of effect.

## 5 Conclusion

In summary, this meta-analysis has demonstrated that Baduanjin significantly improves glucose and lipid metabolism in patients with T2DM. Subgroup analyses suggest that optimal outcomes may be achieved with sessions lasting 40–45 minutes, administered three times per week over a period of 24–48 weeks.

## Data Availability

The original contributions presented in the study are included in the article/**Supplementary Material**. Further inquiries can be directed to the corresponding authors.
