# Supplementary material for: Optimal exercise parameters of Baduanjin for improving glycemic and lipid control in type 2 diabetes: a systematic review and meta-analysis
Source: Front Endocrinol (Lausanne). 2026 Feb 24;17:1731466. doi: 10.3389/fendo.2026.1731466 (PMC12971460; doi:10.3389/fendo.2026.1731466)
Supplement: Supplementary file 2 [file Table2.docx]

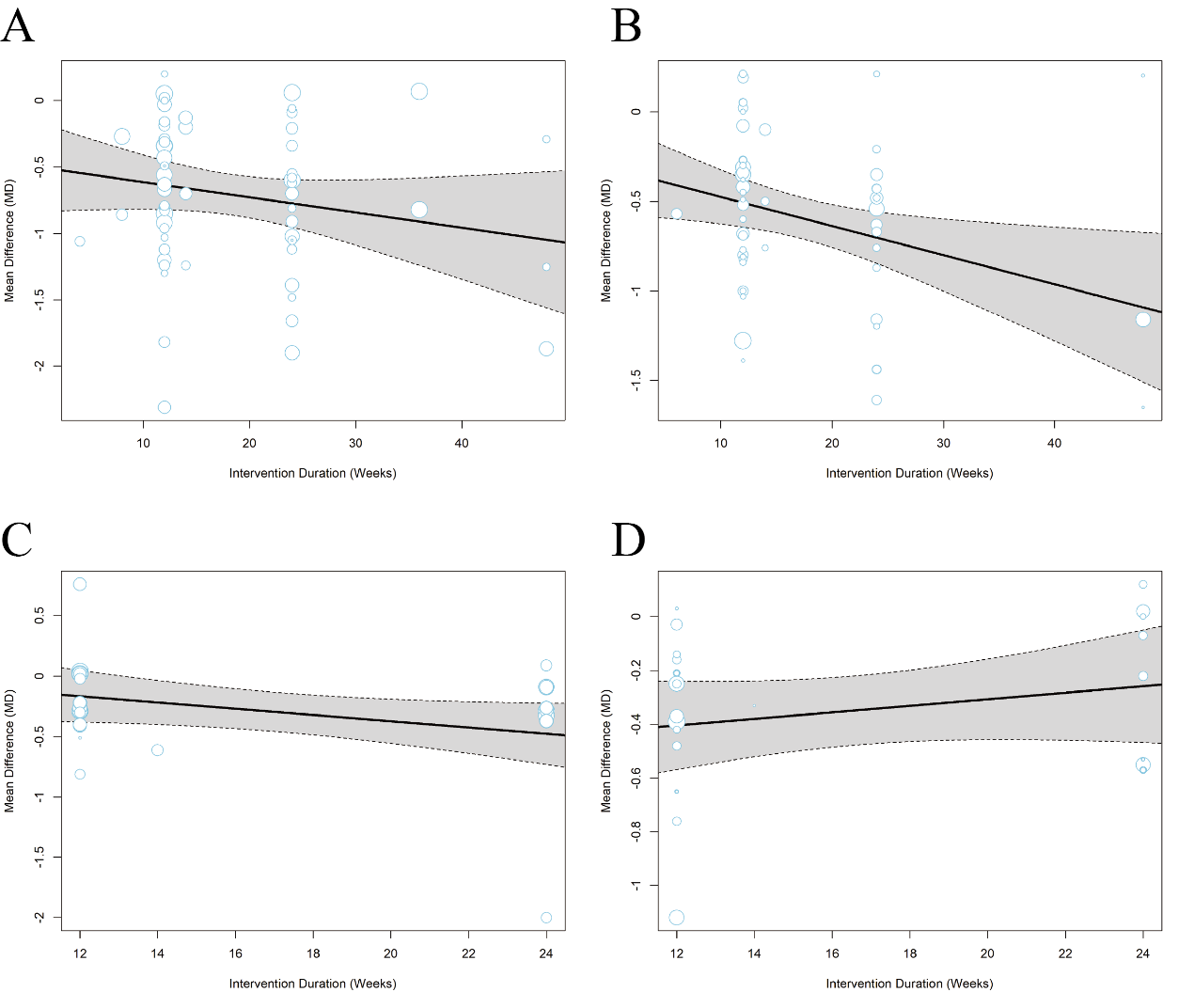


Figure 1. Meta-regression analysis of the association between intervention duration and metabolic outcomes. The bubble plots illustrate the relationship between the duration of intervention (in weeks) and the effect sizes for (A) FBG, (B) HbA1c, (C) TG, and (D) TC.


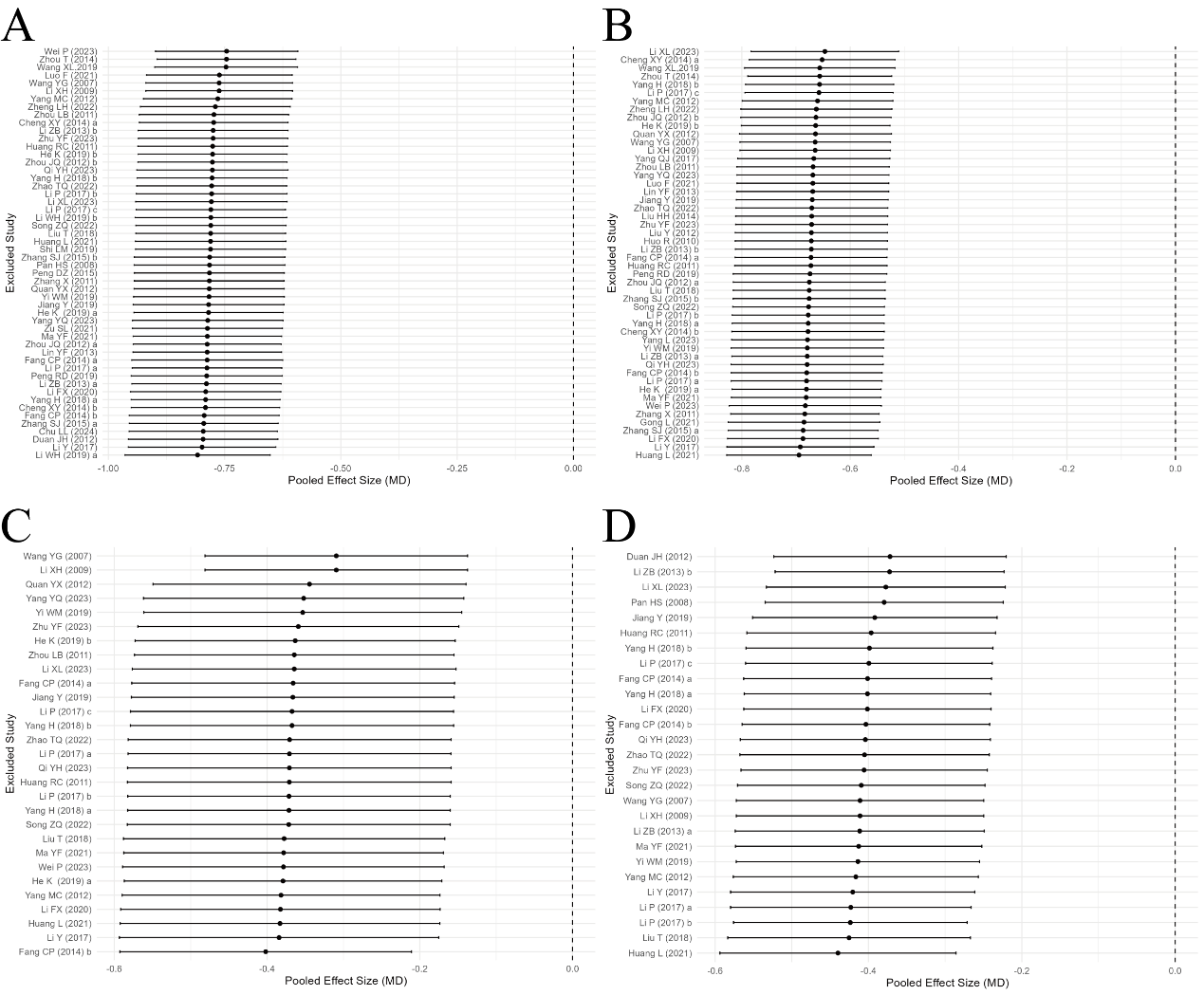


Figure 2. Sensitivity analysis using the leave-one-out method. The plots display the stability of the pooled estimates by sequentially excluding one study at a time for (A) FBG, (B) HbA1c, (C) TG, and (D) TC. The vertical line represents the pooled effect size of all included studies.


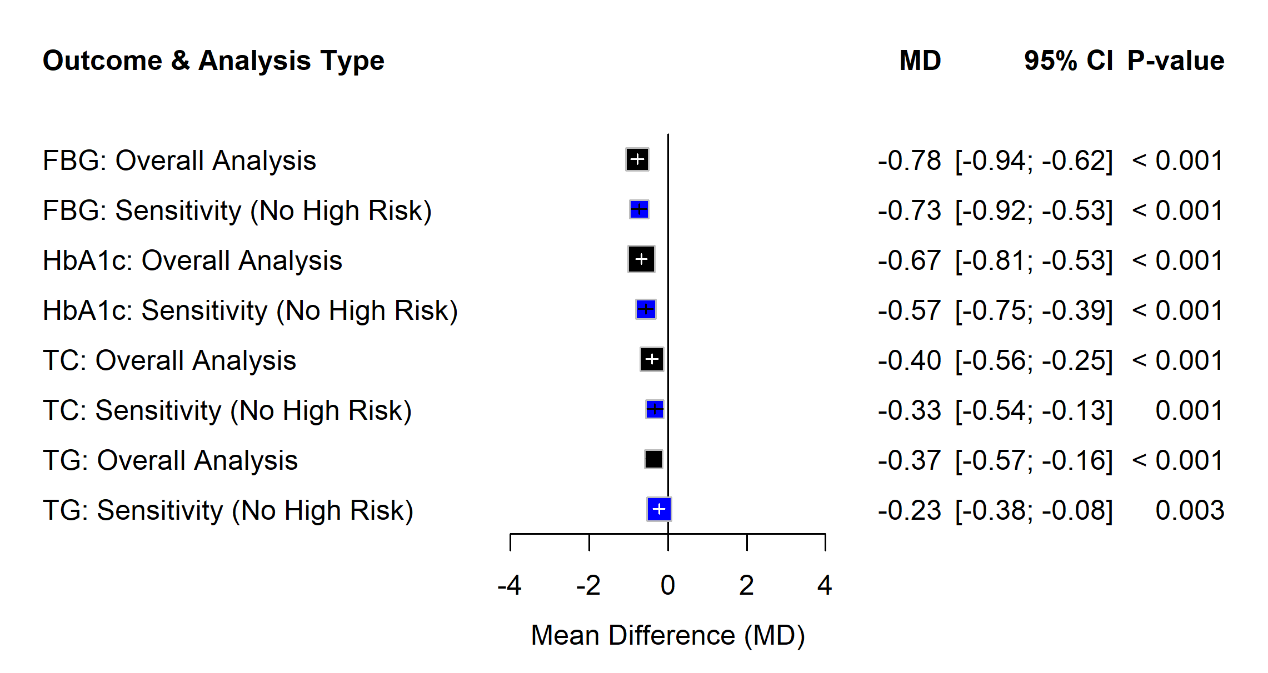


Figure 3. Sensitivity analysis excluding studies with a high risk of bias.


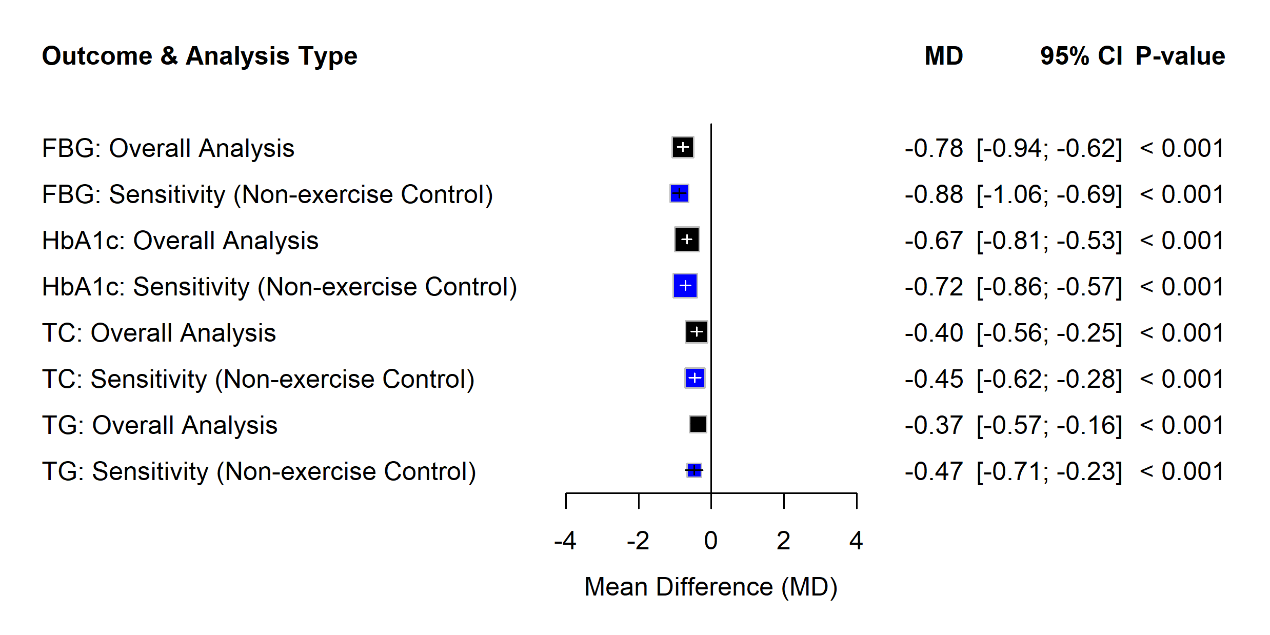


Figure 4. Sensitivity analysis excluding studies utilizing exercise as the control intervention.
